# RNAmute: RNA secondary structure mutation analysis tool

**DOI:** 10.1186/1471-2105-7-221

**Published:** 2006-04-25

**Authors:** Alexander Churkin, Danny Barash

**Affiliations:** 1 Department of Computer Science, Ben-Gurion University, 84105 Beer Sheva, Israel; 2 Genome Diversity Center, Institute of Evolution, University of Haifa, 31905 Haifa, Israel

## Abstract

**Background:**

RNAMute is an interactive Java application that calculates the secondary structure of all single point mutations, given an RNA sequence, and organizes them into categories according to their similarity with respect to the wild type predicted structure. The secondary structure predictions are performed using the Vienna RNA package. Several alternatives are used for the categorization of single point mutations: Vienna's RNAdistance based on dot-bracket representation, as well as tree edit distance and second eigenvalue of the Laplacian matrix based on Shapiro's coarse grain tree graph representation.

**Results:**

Selecting a category in each one of the processed tables lists all single point mutations belonging to that category. Selecting a mutation displays a graphical drawing of the single point mutation and the wild type, and includes basic information such as associated energies, representations and distances. RNAMute can be used successfully with very little previous experience and without choosing any parameter value alongside the initial RNA sequence. The package runs under LINUX operating system.

**Conclusion:**

RNAMute is a user friendly tool that can be used to predict single point mutations leading to conformational rearrangements in the secondary structure of RNAs. In several cases of substantial interest, notably in virology, a point mutation may lead to a loss of important functionality such as the RNA virus replication and translation initiation because of a conformational rearrangement in the secondary structure.

## Background

RNAMute is a user friendly computer tool that analyzes point mutations in the secondary structure of RNAs. Initial ideas can be found in [1] and associated works in the late 80's [2,3]. Since then, much progress has been made in the field RNA secondary structure prediction [4], with the gradual development of sophisticated energy minimization folding prediction packages (most widely used, Zuker's mfold [5] and the Vienna RNA package [6,7]). The possibility of reliably predicting conformational rearranging point mutations in the secondary structure of RNAs has been revisited in [8], suggesting a coarse-grain tree graph representation of the RNA secondary structure [2] and the use of mathematical theorems that relate to eigen-decomposition of the Laplacian matrix [9,10] corresponding to the coarse-grain tree graphs. Both fine-grain and coarse-grain graph representations, including distance measures between the graphs, have been implemented in the Vienna RNA package [6]. We use the Vienna RNA package as the core of RNAMute, attaching to it the mutation prediction procedure described in [8]. To initially test the approach, experimental results from [11,12] were taken. Motivation for the use of RNAMute can be found in the literature [13-16]. These constitute example cases in which point mutations that affect the functionality of an RNA molecule cause a conformational rearrangement in its secondary structure, as explained in detail in the final Section.

## Implementation

### Availability

The package can be downloaded from [17]. After downloading, extract the file with the commands:

1. >gunzip RNAMute.tar.gz

2. >tar xvf RNAMute.tar

More details on how to run the program are contained in the ReadMe.html file.

### The package content

1. mute_single – performs all possible "single point mutations" in an RNA sequence. The mute_single routine predicts the secondary structure of the wild type and mutants using Vienna's RNAfold, then calculates several different representations and similarity measures between the wild type and mutants, and finally produces a "result" file from the results obtained.

2. RNAmute.java – the main routine. Creates a "friendly" interface for the user. Receives as input a file with an RNA sequence, runs "mute_single", and generates an HTML file called "RESULT_TABLE.html" that contains all the processed data from the "result" file organized in various tables.

3. calcEig2 – calculates the second smallest eigenvalue of the Laplacian matrix for each single point mutation.

4. b2Shapiro – converts the full structure from bracket notation to the weighted coarse grained notation introduced by Bruce Shapiro. This routine uses a function that is located in the Vienna package's "lib" directory.

5. runRnaMute – similar to RNAmute, but enables the user to insert the RNA sequence in a text area of the GUI instead of using a file.

Programs taken from the Vienna RNA package:

1. RNAfold – predicts minimum energy secondary structures and base pairing probabilities.

2. RNAdistance – calculates the distance between two RNA secondary structures represented as dot-bracket strings.

The package also contains the source code for all its components.

While the program runs, a new directory called "htmlDir" will be created. This directory contains all the HTML pages and all the drawings of the RNA secondary structures that are being calculated.

### Preparation and compilation

RNAMute is currently available on a Linux platform, therefore all preparations and compilations that will be mentioned should be performed on a Linux platform with Java and "GNU CC" compiler installed. RNAMute has all its components already compiled and may be used without any compilations, although it has some components written in C that in some architectures may not work. In such a case, the Vienna RNA package should be downloaded from the website [18] and directory "ViennaRNA-1.4\lib" should be compiled by running the command "make" in this directory. All files from the directory "RNAMute\RNAMute_progs" should be copied to "ViennaRNA-1.4\Progs" and compiled with "makefile". "Makefile" that appears in the "ViennaRNA-1.4\Progs" directory should be overwritten. After the compilation finishes, files: "b2Shapiro", "calcEig2", "RNAdistance", "RNAfold" and "mute_single" should be copied from the "ViennaRNA-1.4\Progs" directory to the "RNAMute\bin" directory. All files that are already in the aforementioned directory should be overwritten. The user should then make sure that all files in the "RNAMute\bin" directory are in an executable mode. If not, it is possible to change their mode by typing the command: >chmod 700 file_name, where file_name is each file from the list above.

## Results and discussion

**Figure 1:**
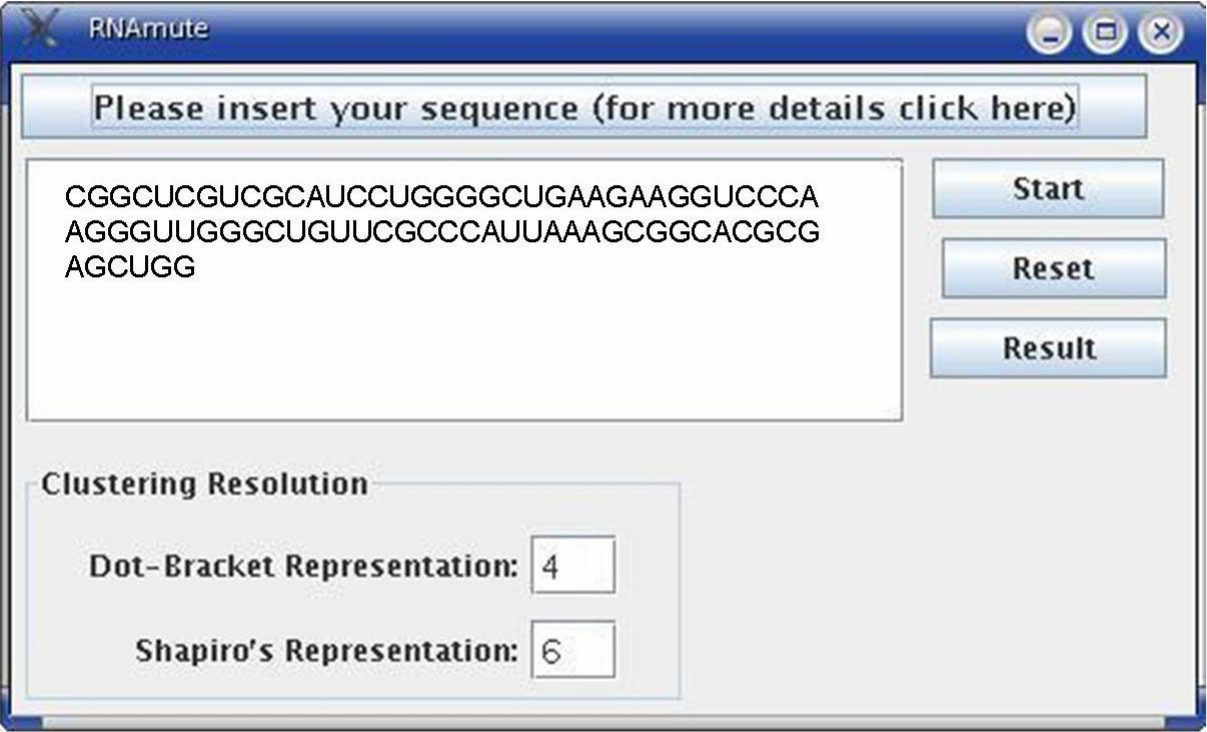
**RNAMute Input Screen**. Initial Java GUI for providing the RNA sequence that the user would like to analyze.

**Figure 2:**
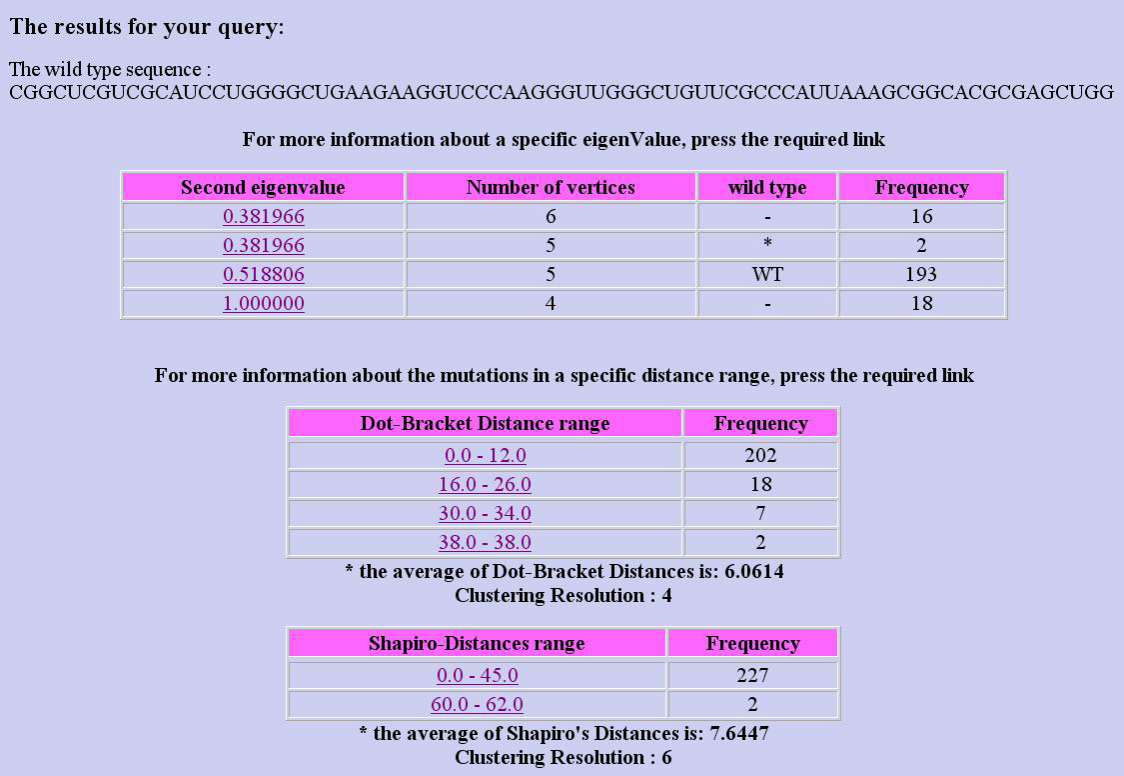
**Categorization Tables**. Main HTML page for the categorization of single point mutations according to the mutants secondary structure similarities relative to the wildtype structure.

**Figure 3:**
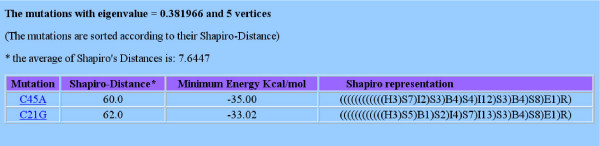
**Mutations Descriptor Data**. Mutations descriptor HTML page. For each category, the single point mutations belonging to each category are listed along with their minimal free energy and string representation.

**Figure 4:**
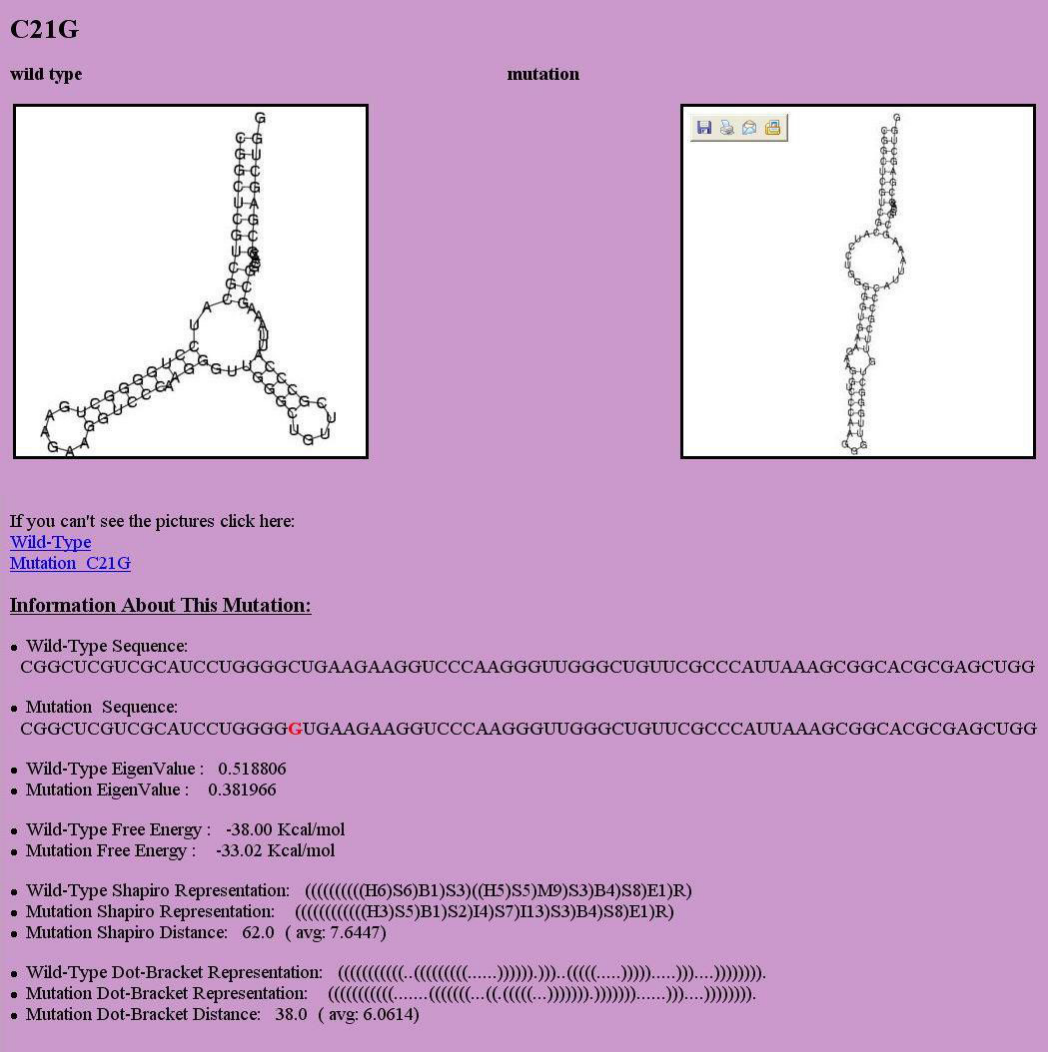
**Mutant vs. Wildtype Structure and Energy Information**. For each single point mutation, relevant secondary structure and energy information is listed along with a graphical drawing for both the mutant and the wildtype. This allows a direct comparison between the corresponding mutant and the wildtype structure.

The input to RNAMute is simply an RNA sequence (see Figure 1). Subsequently, after pressing the "Start" button, RNAMute scans all possible single point mutations in that sequence and computes their folding prediction using Vienna's RNAfold program. The analysis of point mutations is illustrated in Figures 2, 3 and 4 and will be described in detail in the manual document file included in the package. Such an analysis is capable of predicting conformational rearranging single point mutations, for example the point mutation that is responsible for switching between FORM 1 WT RNA and FORM 2 M3 RNA as described and examined experimentally in [11]. Results can be observed by pressing the "Result" button. An HTML page with three tables will appear (Figure 2). For illustration, we use the IV domain piece that was cut from rRNA of the *Tetrahymena thermophila *[12].

The first table in Figure 2 divides all new structures that were predicted from all point mutations to groups according to their second eigenvalue of the Laplacian matrix [8]. This table also shows how many vertices the structure in each group contains, and the number of structures in each group. In the third column, a group that holds the wild type is marked with "WT", and groups that have the same number of vertices as the WT are marked with "*". The user can click on each value in the first column to view the list of mutations with this value and the specified number of vertices. For example, clicking on eigenvalue 0.381966 (with 5 vertices) will open the table shown in Figure 3. This table contains: (1) mutation's names. (2) distances of the mutations from the WT according to Shapiro's representation for both the mutation and the WT. Mutations in this table are sorted by this column. (3) Minimum Energy (in Kcals/mol) of the secondary structure of mutated sequence. (4) the Shapiro representation of the mutated sequence. Additional information about each mutation can be obtained by clicking on the mutation name. Figure 4 shows the HTML page with additional information for mutation C21G that contains: drawings of RNA secondary structures for the WT sequence and mutated sequence; option to download both drawings in ps format; WT sequence and the mutated sequence; the eigenvalue of the WT secondary structure and of the mutant secondary structure; the WT's free energy and the mutant free energy (in Kcals/mol); Shapiro and dot-bracket representations of both the WT and mutant; distances (according to Shapiro and dot-bracket representations) of mutant from the WT, and the average Shapiro and dot-bracket distances of all mutants.

The second table in Figure 2 divides structures to groups according to their "Dot-bracket distance" from the wild type structure. This distance is calculated between the dot-bracket representations of WT and mutations. The first column contains the distance's ranges that were calculated according to "clustering resolution" for "dot-bracket representation", which is set to 4 by default, and can be changed by the user. Clustering resolution of X means that distances are sorted in each group and if there are two distances such that the difference between them is less than X, these distances are in the same group.

The user can click on a specific distance range in the first column to observe the list of mutations with a distance in this range. For example, distance range of 38.0-38.0 has a similar table as in Figure 3 and has only 2 mutations. This distance range is interesting to explore because it contains structures of mutations with a relatively large dot-bracket distance from WT. Additional information about each mutation in each table can be obtained by pressing on the mutation name, such as in Figure 3 and the information page that will be obtained as depicted in Figure 4. In our case these are the same two mutations as were obtained from the first table (eigenvalue 0.381966) and these are the only mutations in the run that break one of two hairpins and linearize the structure.

The third table in Figure 1 is similar to the second table but it groups structures according to their Shapiro distance which is obtained from the Shapiro representation of the WT and mutation's structure. It is possible to see that the third table also groups two mutations with a relatively large distance to a separate category, and these two mutations are exactly the same mutations that were found in "Eigenvalue table" and "Dot-bracket table".

From the illustrated example we can conclude that the RNAmute package was able to find mutations that change the secondary structure of the wildtype and it divided these mutations into separate categories in all tables. In the first table these mutations fall to the category with specific second smallest eigenvalue of the Laplacian matrix corresponding to the coarse-grain tree graph representation; in the second and the third tables these mutations fall into categories with largest distances.

## Conclusion

**Figure 5:**
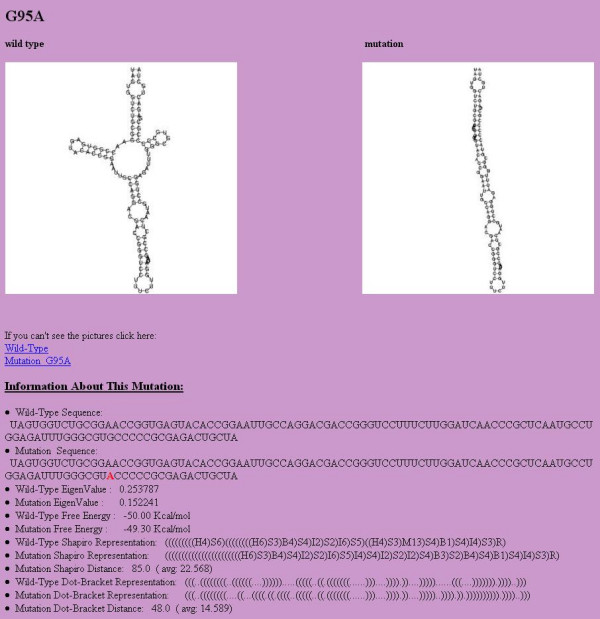
**Single Point Mutation Prediction in the 5'UTR ofHCV by RNAMute**. A successful prediction by RNAMute, illustrating its potential capability to detect biologically meaningful findings. The G235A point mutation (corresponding to G95A using our indexing scheme) in the 5'UTR of HCV [16] is predicted by RNAMute to cause a conformational rearrangement. In turn, it is reported to display a dramatic reduction in translation initiation. However, in that reference [16] based on simple base pairing considerations, it was stated that this mutation alters only the primary sequence. With the availability of RNAMute, alterations in the secondary structure can easily be detected.

In examining its biological relevance, RNAMute can be used in predictions and analyses related to mutagenesis experiments. For example, in [13] it was shown that individual point mutations are capable of inactivating spectinomycin resistance in *Escherichia coli *and secondary structure predictions displayed conformational rearrangements. Moreover, in examples where the sequences examined contain less than 100 nt, virologists have shown interest in computerized predictions of mutations that disrupt the stable stem-loop structure that characterizes Hepatitis C Virus (HCV) [14-16]. Such structural changes may lead to alterations in virus replication [14,15] or translation initiation [16]. In the latter reference [16], the single point mutations A172G, G229A, and G235A were found to display a dramatic reduction in translation initiation in site-specific mutagenesis experiments affecting the stem-loop IIIc. While it was obvious that A172G and G229A disrupt the base pairing required to form the structures in and around stem-loop IIIc, G235A was assumed to only alter the primary sequence since no obvious Watson-Crick base pairing modifications appear at first glance. However, using RNAMute, G235A can be found to disrupt the important stem-loop structure as well (Figure 5), where G95A according to our indexing scheme corresponds to G235A in the indexing scheme used in [16]. In Figure 5, we only used a segment of the HCV RNA as our initial sequence to RNAMute after verifying that the wildtype of the segment is accurately predicted by mfold and Vienna's RNAfold. Thus, with the public availability of RNAMute, computational mutation predictions that are needed to detect novel functional biological findings can be improved.

## Availability and requirements

**Project name: **RNAMute

**Project home page: **

**Operating system(s): **web access: not applicable, stand-alone: LINUX

**Programming language: **C, Java

**Other requirements: **stand alone:Java 1.4.0 or higher, GNU CC compiler

**License: **None

**Any restrictions to use by non-academics:** None

## Authors' contributions

DB conceived the study, coordinated and participated in software design and drafted the manuscript. AC worked on software design, carried out development and impelementation, and participated in drafting the manuscript.
